# The Impact of Reprocessing with a Quad Screw Extruder on the Degradation of Polypropylene

**DOI:** 10.3390/polym14132661

**Published:** 2022-06-29

**Authors:** Mansour Alotaibi, Thamer Aldhafeeri, Carol Barry

**Affiliations:** Department of Plastics Engineering, University of Massachusetts Lowell, Lowell, MA 01854, USA; mansour_alotaibi@student.uml.edu (M.A.); thamer_aldhafeeri@student.uml.edu (T.A.)

**Keywords:** polymer degradation, twin-screw extruder, quad-screw extruder

## Abstract

During mechanical recycling, polypropylene typically is reprocessed using a single- or twin-screw extruder. The degradation of polypropylene during this reprocessing reduces the polymer’s molecular weight and, consequently, limits the performance of the recycled resin. This work investigated the impact of a quad screw extruder (QSE), which has greater free volume, on the reprocessing of an impact copolymer polypropylene. To mimic the recycling process, the polypropylene was subjected to three processing cycles using a QSE and a comparable twin-screw extruder (TSE) operated at three screw speeds. The reprocessed materials were characterized for their rheological, morphological, and mechanical properties. For both extruders, increasing the number of reprocessing cycles and the screw speed resulted in higher melt flow indices, decreases in zero-shear viscosity, and shifting of the crossover points for the storage and loss moduli, which indicate reductions in the molecular weight and narrowing of the molecular weight distribution of the polypropylene. The QSE exhibited greater reductions in molecular weight compared to the TSE, probably due to the higher stresses associated with the three intermeshing points along its screws. Reprocessing caused a significant reductions in the Izod impact strength of the reprocessed polypropylene, which correlated with reductions in the particle size and particle size distribution of the dispersed rubbery phase in the polypropylene during reprocessing.

## 1. Introduction

Polypropylene (PP) is the second most commonly used polymer after polyethylene. It is available in three families: homopolymer polypropylene (HPP), random polypropylene (RCP), and impact copolymer polypropylene (ICP). In view of its ease of processing, low density, good chemical resistance, and low cost, polypropylene is suitable for use in a wide range of applications, including packaging, consumer goods, building and construction, and automotive [1,2]. The usage of polypropylene, however, has significantly increased in recent decades, creating in more post-consumer waste, especially for disposable products and packaging. As a result, several regulations by governments around the world require the reduction of the waste polypropylene [3,4].

Mechanical recycling is the most common technique for reducing waste polymer, and its use has increased due to economic and environmental benefits. This mechanical recycling consists of collecting and sorting the waste, grinding the waste, and then reprocessing the waste [5]. Depending on the level of additives and reinforcements required for the polypropylene, reprocessing is usually performed with a single-screw extruder or a co-rotating twin-screw extruder [5]. Generally, the reprocessing of polypropylene will produce degradation and result in a significant deterioration of the material properties when compared to virgin material; this thermomechanical degradation is due to shear and heat applied during reprocessing [6,7,8]. During reprocessing, chain scission is one of the major degradation pathways for polypropylene [9]. This degradation process occurs mainly because the β-scission of tertiary carbons generates double bonds and free radicals; these entities react with oxygen to produce peroxides and hydroperoxides, which then create more radicals, making the degradation an autocatalytic process [10,11]. The chain scission reactions produce decreases in polymer molecular weight, leading to decreases in melt viscosity and increases in melt flow rate [9,12,13,14]. The reduction in molecular weight negatively impacts the mechanical, structural, and thermal properties and so reduces the uses of recycled material [3,7,15,16]. Therefore, down-cycling, i.e., use of the recycled polymer for structurally weaker products, is one of the challenges in the mechanical recycling of polypropylene [5].

Studying multiple-extrusion history has been found to be a very useful method to simulate mechanical recycling by melt processing [17,18] and to evaluate material processing stability and estimate product properties [18,19]. Several studies of polypropylene have been conducted with co-rotating twin-screw extruders, single-screw extruders, and injection molding machines [7,9,10,12,13,14,16,17,18,20,21,22,23,24,25]. These studies have shown that the degradation during reprocessing is a function of polymer structure, processing method and processing conditions (i.e., temperature, screw speed, screw configuration, and output), and antioxidant efficiency. Costa et al. [12] demonstrated that the polypropylene was degraded by scission chain during multiple cycles of processing at different melt temperatures in a single-screw extruder. In addition, they observed that the degradation increased with increasing melt temperature and the number of cycles, resulting in greater molecular weight reductions of the polymer. Using a twin-screw extruder, Martínez-Jothar et al. [14] studied the effect of processing temperatures (240, 260, and 280 °C) during reprocessing of polypropylene. They found that the reprocessed materials presented higher carbonyl indices, higher melt flow indices, and lower average molecular weights when the number of cycles and the melt temperature were increased. When Bouaziz et al. [17] investigated the reprocessing of polypropylene at different screw rotation speeds (300, 800, and 1200 rpm) using a twin-screw extruder, they reported that degradation of polypropylene increased with higher screw speeds and a greater number of cycles. Canevarolo [22] investigated the impact of screw design on the degradation of polypropylene with a twin-screw extruder. He examined two screw designs; the first one had only conveying elements, whereas the second one had kneading blocks. He found that the screw configuration with a set of kneading blocks resulted in greater degradation. After investigating the effect of single- and twin-screw extruders on the degradation of contaminated post-consumer polypropylene, Garcia et al. [10] concluded that the materials reprocessed using single-screw extruders exhibited a lower decrease in molecular weight.

The general consensus was that degradation of polypropylene during reprocessing can be reduced by lowering the barrel temperature profile and reducing the screw speeds. In addition, more effective stabilization packages and greater concentration of stabilizers can help to prevent polypropylene from degrading and improve recyclability [3,5,18,26]. The most common antioxidant systems are phenolics and hindered amines [3,5].

In contrast to co-rotating twin-screw extruders, quad-screw extruders (QSEs) have not been investigated for to reprocessing of polymers. A quad-screw extruder consists of four co-rotating parallel fully-intermeshing screws. Compared to twin-screw extruders, QSEs have greater free volume, which has produced greater throughputs and lower shear rates [27]. The three intermeshing regions of the screws provide high shear stress and increase the area available for kneading action on the melt; this feature has produced better mixing compared to twin-screw extruders [27].

Therefore, the objective of this work was to evaluate the impact of a QSE on the recyclability of polypropylene. The virgin impact copolymer polypropylene was subjected to multiple reprocessing cycles at several screw speeds in a QSE and compared with the performance for a similar TSE. The effect of reprocessing was assessed by extruder parameters (melt temperature and head pressure) as well as the melt flow index, rheological properties, impact strength, and morphology of the reprocessed polypropylene.

## 2. Experimental Method

### 2.1. Material

A commercial grade of a heterophasic polypropylene copolymer supplied by Borealis (BB125MO, Belgium) was used for this work. The reported melt-flow index was 1.3 dg/min (230 °C/2.16 kg). The heterophasic copolymer consists of a homopolymer polypropylene matrix with a dispersed rubbery copolymer phase (ethylene propylene rubber).

### 2.2. Processing Methods

The reprocessing of the polypropylene resin was carried out using a co-rotating twin-screw extruder (TSE) from Technovel Corporation (model: KZW15TW-45/60 MG-NH, Osaka, Japan) and a quad-screw extruder (QSE) from Technovel Corporation (model: WDR15QD-45MG-NH, Osaka, Japan). Both extruders had 15 mm diameter screws with length-to-diameter ratios of 45:1. Moreover, both extruders had the same screw configuration. The screw configuration contained only one set of 90° kneading blocks and two sets of 45° forward-kneading blocks immediately after the first feed port to help to melt polymer; the rest of the screw consisted of conveying elements. This screw design was used to reduce the shear on the polymer. The barrel temperature profile was set to 200–230 °C from the first feed section to the strand die. The extrudate was cooled using a water bath, dried by pressurized air, and then strand-pelletized by a rotary cutter. The feed rate was held constant at 2.3 kg/h because it was the maximum feed rate tolerated by the TSE. The reprocessing was performed at screw speeds of 500, 1000, and 1500 rpm. For each screw speed, all samples were recycled three times at the same processing conditions. The melt temperature and head pressure were collected from the machines’ readouts to evaluate machine response. The melt temperature was measured using a type K thermocouple (Dynisco, Franklin, MA, USA) protruding 6 mm into the melt stream, whereas the head pressure was measured using a pressure transducer (Dynisco, Franklin, MA, USA) located before the die.

### 2.3. Characterization

#### 2.3.1. Melt Flow Index

The melt flow index (MFI) measurements were performed using an extrusion plastometer (Dynisco, model: 710-1-5-010-14, Franklin, MA, USA). ASTM D1238-20 (Procedure A) was followed with the temperature set at 230 °C and weight load at 2.16 kg. For each reprocessing condition, five measurements were carried out, and the average values were calculated. The relative melt flow indices (MFI/MFI_o_), i.e., ratios of the reprocessed samples’ average MFIs to virgin sample’s average MFI, were reported.

#### 2.3.2. Rheological Properties

The rheological properties of the reprocessed samples were determined using a parallel plate rheometer (TA Instruments, model: ARES-G2, Newcastle, DE, USA). The specimen disks were prepared using micro-injection molding (Xplore Instruments BV, Sittard, The Netherlands) at a temperature of 230 °C and soak time of 3 min. The parallel plates had a diameter of 25 mm and gap of 1.5 mm; the set temperature was 230 °C. For these measurements, a strain sweep was first performed over strain range of 0.1 to 100% and at fixed frequency of 10 Hz to determine the range of linear viscoelastic behavior of the polypropylene. Then, frequency sweep measurements were performed for a frequency range of 0.02 to 15.92 Hz and at a constant strain of 2%, which was within the region of linear viscoelastic region for the polymer.

To determine the zero-shear viscosity (η_o_), the non-linear curves of complex viscosity as function of frequency were fitted to the Cross model [28] using Trios software (TA Instruments). The zero-shear viscosity can be related to the weight average molecular weight (M_w_) using [10]:

(1) $$\eta_{o}\;=k(M_{w}{)}^{a}$$

where a = 3.4, and k is constant, that is, the same for all samples. Therefore, the molecular weight ratio (Mw_1_/M_W2_) was calculated as:

(2) $$\frac{M_{w1}}{M_{w2}}=\sqrt[{a}]{\frac{\eta_{o1}}{\eta_{o2}}}$$

where M_W1_ is the molecular weight of the unprocessed material, M_W2_ is the molecular weight of the reprocessed material, η_o1_ is zero-shear viscosity of the unprocessed material, and η_o2_ is zero-shear viscosity of the reprocessed material.

**Figure 1:**
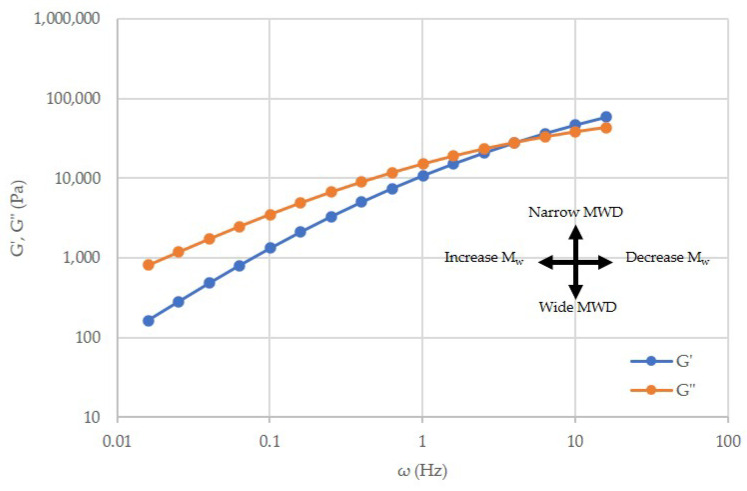
G’ and G” for unprocessed polypropylene, showing the crossover point where G’ = G”; modeled after [10,26].

Storage modulus (G’) and loss modulus (G”) curves also were obtained from the parallel plate rheology. These curves were analyzed by the shift in crossover points (G*) of the storage modulus (G’) and loss modulus (G”) curves. As shown in Figure 1, the shift in crossover point to lower or higher angular frequencies implies that the molecular weight has changed. In contrast, shifts in the crossover point to lower or higher moduli values suggested that the molecular weight distribution (MWD) has been altered.

#### 2.3.3. Morphology

The molded specimens of each sample were examined by field-emission scanning electron microscopy (FESEM) using a JEOL, model: JSM 7401F (Seoul, Korea). The accelerating voltage was maintained constant at 3 kV. Prior to imaging, the injection-molded samples were fractured under liquid nitrogen to avoid any disturbance to the molecular structure and then etched with xylene at room temperature for 24 h to extract the elastomeric (ethylene propylene rubber) phase. Then, the etched samples were coated with platinum for 120 s using a vacuum sputter coater (Leica, model: SCD500, Deerfield, IL, USA).

#### 2.3.4. Izod Impact Test

Notched Izod impact testing was performed using an Izod Impact Tester (TMI Testing Machines, model: 43-1) and a 1.36 J. pendulum. In accordance with ASTM D256-10, the impact properties were tested at room temperature and 40% relative humidity. Prior impact testing, the specimens were molded using Arburg, model: Allrounder 320, injection molding machine (Loßburg, Germany) operated at 230 °C. More than five measurements were performed for each reprocessed sample, and the average values were reported.

## 3. Results and Discussion

### 3.1. Extrusion Parameters

**Figure 2:**
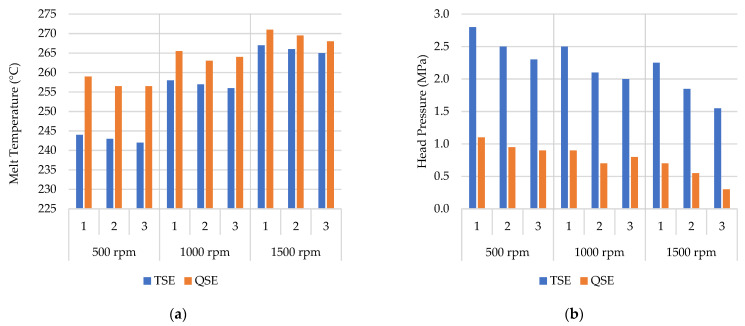
The effect of screw speed and reprocessing cycle (1–3) on the (**a**) melt temperature and (**b**) head pressure developed in the TSE and QSE.

Figure 2a illustrates the effects of screw speed and cycle number on the melt temperatures in the TSE and the QSE. All melt temperatures were greater than the set temperature of 230 °C, and only some of these increases were due to shear heating along the protruding melt thermocouple. For both machines, the melt temperatures increased linearly with increases in screw speed. These increases indicate that the mechanical work associated with the higher screw speeds increased the levels of shear and made the melt temperature much higher than expected. The findings are consistent with prior work that has shown increases in melt temperature with increasing screw speed [17,27]. Additionally, the melt temperature decreased slightly with a higher number of reprocessing cycles. This decrease was attributed to the polymer degradation, which reduces in the melt viscosity and produces less shear heating of the polymer. The QSE, however, generated higher melt temperatures as compared to the TSE. These increases were attributed to the high shear stresses associated with the three screw intermeshing regions in the QSE; these results were consistent with prior comparisons of the QSE with a TSE for mixing [27].

In both extruders, the head pressure decreased as the screw speed and number of reprocessing cycles increased (Figure 2b). The decrease in head pressure was associated with the decrease in melt viscosity and indicated a decrease in molecular weight. This behavior was in agreement with other authors’ findings for TSEs [9,27]. The QSE also exhibited significantly lower head pressures than the TSE. These lower pressures were consistent with those from a prior study [27] and were attributed to the greater free volume of the QSE.

### 3.2. Melt Flow Index

**Figure 3:**
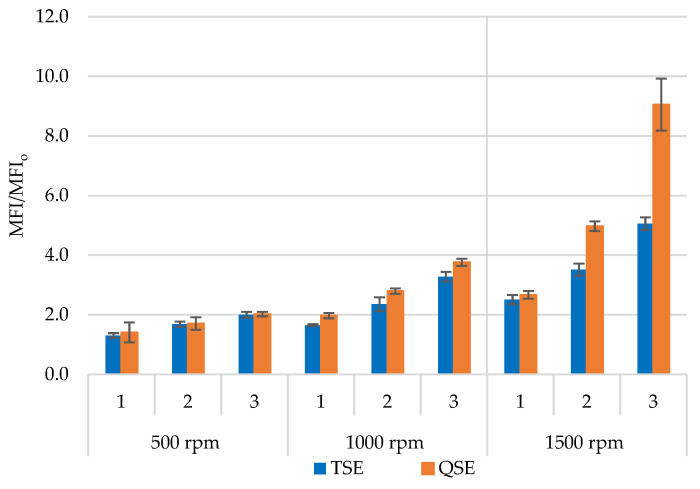
Melt flow index ratio (MFI/MFI_o_) for polypropylene reprocessed using the TSE and the QSE at three screw speeds and for three reprocessing cycles.

The melt flow index (MFI) provides essential information regarding the material’s flowability because it indicates the polymer’s molecular weight and viscosity. Figure 3 presents the effect of screw speed and number of reprocessing cycles on the relative melt flow index (MFI/MFI_o_). As expected, there was a substantial increase in MFI with the number of cycles and the screw speed due to the thermomechanical degradation of the polypropylene. Over the three processing cycles in the TSE, the MFI/MFI_o_ increased from 1.3 to 2.0, 1.7 to 3.3, and 2.5 to 5.1 for screw speeds of 500, 1000, and 1500 rpm, respectively. These results were in agreement with other authors’ work on polypropylene homopolymers and copolymers reprocessed using single- and twin-screw extruders [9,13,17,18]. At a screw speed of 500 rpm, the QSE produced increases in MFI/MFI_o_ that were similar to those of the TSE. Slightly larger increases in MFI/MFI_o_ (2.0 to 3.8) occurred for a screw speed to 1000 rpm in the QSE. With the highest screw speed, however, the QSE exhibited a significant increase in MFI/MFI_o_ (2.7 to 9.0) with a greater number of reprocessing cycles. These results suggest that QSE produced more degradation at higher screw speeds (>1000 rpm); this finding was consistent with prior work on mixing, which indicated that high screw speeds in the QSE produced greater material degradation [27].

### 3.3. Rheology

The storage modulus (G’) represents the elastic behavior and the amount of stored energy, whereas the loss modulus (G”) characterizes the amount of dissipate energy. The changes in G’ and G” with frequency reflect the mobility of the polymer chains in bulk [12]. The crossover points (G*) are where the storage modulus is equal to the loss modulus. The crossover points (G*) and frequencies (ω*) for polypropylene reprocessed using the TSE and QSE are listed in Table 1; for some samples, crossover points were not observed and may have occurred at a higher frequency than the test frequency range. For the unprocessed polypropylene, G* was 27.8 kPa, and ω* was 4.0 Hz. In the first reprocessing cycle with a screw speed of 500 rpm, the crossover points and frequencies were increased to 29.4 kPa and 5.6 Hz in the TSE and to 28.5 kPa and 6.2 Hz in the QSE. These results suggest that the QSE produced a greater reduction in molecular weight and a narrower molecular weight distribution than the TSE for the first reprocessing cycle. With more reprocessing cycles at 500 rpm, the G* and ω* values increased, but they were not always higher for the QSE. When the screw speed was 1500 rpm, the crossover points and frequencies increased further and reached 32.7 kPa and 9 Hz in the TSE and 33 kPa and 11.6 Hz in the QSE after the first reprocessing cycle. At 1000 and 1500 rpm, the G* values did not change significantly with a greater number of reprocessing cycles and appeared to be similar for the TSE and QSE. In contrast, the ω* values for screw speeds of 1000 and 1500 rpm increased with screw speed, increased with the number of reprocessing cycles, and were greater for the QSE than the TSE. This behavior at the higher screw speeds suggests than the molecular weight distribution was not changing significantly but that the molecular weight was further reduced by higher screw speeds and greater numbers of reprocessing cycles and that it was greater for the QSE than the TSE. Samples without observable crossover points showed noticeable viscous behavior (i.e., a higher contribution of G”), indicating extensive degradation when the polypropylene material was exposed to further reprocessing cycles at higher screw speeds. Overall, these findings indicate that the higher shear stresses imposed by the QSE caused a greater reduction in molecular weight and a narrower molecular weight distribution.

**Figure 4:**
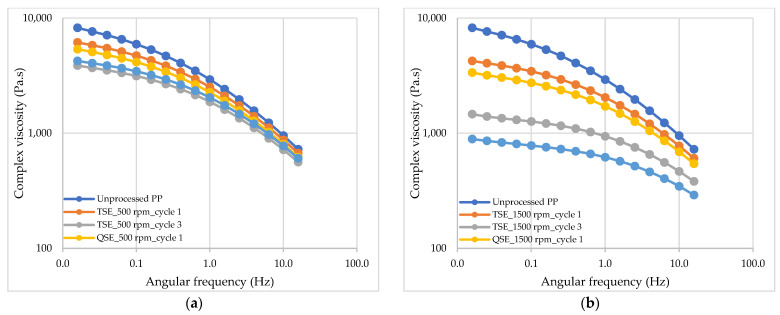
The effect of screw speed and reprocessing cycles on complex viscosity as function of angular frequency of PP material reprocessed by TSE and QSE: (**a**) at 500 rpm and (**b**) at 1500 rpm.

As shown in Figure 4, the complex viscosity of the polypropylene also was affected by reprocessing. In comparison to the unprocessed polypropylene, increasing the screw speed and number of reprocessing cycles in the TSE and QSE resulted in a decrease in the overall viscosity. At a screw speed of 500 rpm, the viscosity of polypropylene reprocessed with both the TSE and QSE showed similar complex viscosity–frequency curves as the unprocessed polypropylene (Figure 4a). The viscosity decreased slightly with each reprocessing cycle and was slightly greater for the QSE. When the screw speed was increased to 1500 rpm, however, there was a significant decrease in complex viscosity with each reprocessing cycle and a much greater reduction in complex viscosity for the QSE (Figure 4b). These findings indicate that the degree of degradation increased with screw speed and caused a significant reduction in molecular weight. The complex viscosity–frequency curves also showed more noticeable lower Newtonian plateaus, an indication of lower molecular weights and narrow molecular weight distributions in the materials (Figure 4b). The greater reduction in complex viscosity with the QSE was primarily due to the higher shear stresses in the QSE.

The complex viscosity–frequency curves did not exhibit a clear lower Newtonian plateau (Figure 4). To predict the zero-shear viscosity over a lower range of frequencies, it was necessary to extrapolate data through appropriate model fitting in order. The data of complex viscosity versus frequency were fitted using a Cross model, an empirical model that is used to fit non-Newtonian data over a wide range of shear rates [28]. In the Trios package, the coefficients of determination (R^2^) for these fits were 0.9999. Since these values were very close to 1.0, the data were a good fit for the Cross model. Therefore, the calculated zero-shear viscosities could be used to analyze the impact of reprocessing on the polypropylene’s molecular weight.

**Figure 5:**
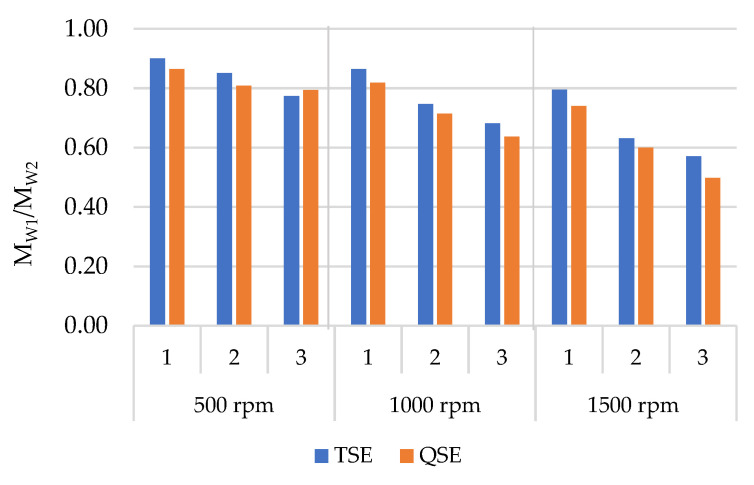
Molecular weight ratio (M_W1_/M_W2_) for polypropylene reprocessed in a TSE and a QSE using three screw speeds and three reprocessing cycles.

Figure 5 presents the molecular weight ratio (M_W1_/M_W2_) of the reprocessed polypropylene samples as a function of the reprocessing cycle in the TSE and QSEs and at different screw speeds; M_W1_/M_W2_ was calculated using Equation (2). The molecular weight ratio was used to quantify the reduction of the molecular weight due to the chain session reactions. In general, the molecular weight ratio for the reprocessed samples decreased with increasing screw speed and the number of reprocessing cycles. For the TSE, these molecular weight reductions were consistent with prior work that attributed reductions in molecular weight to chain scission during reprocessing processes [10,12,27]. Moreover, the QSE generally produced greater reductions in molecular weight than the TSE. At a screw speed of 500 rpm, the molecular weight ratio decreased from 0.90 to 0.77, respectively, for one to three reprocessing cycles in the TSE. In the QSE at the same screw speed, the molecular weight ratios exhibited a decrease of 0.86 to 0.79 with an increasing number of cycles. With a higher screw speed of 1500 rpm, the molecular weight ratio decreased from 0.80 to 0.57 for three reprocessing cycles in the TSE, whereas the molecular weight ratio was reduced from 0.74 to 0.50 during reprocessing in the QSE. These findings were consistent with the melt flow index and complex viscosity results (Figure 3 and Figure 4). The greater molecular weight reduction in the QSE compared to the TSE also suggested that the greater shear stress in the QSE produced greater chain scission.

### 3.4. Izod Impact Strength

**Figure 6:**
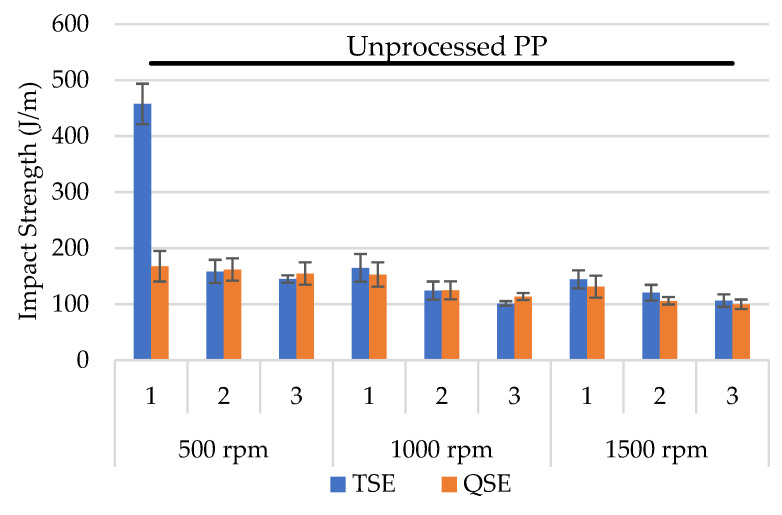
Notched Izod impact strength for polypropylene reprocessed using a TSE and a QSE with three screw speeds and three reprocessing cycles.

As shown in Figure 6, the notched Izod impact strength decreased with reprocessing. With one heat history in injection molding (unprocessed PP), the notched Izod impact strength was 530 J/m. With reprocessing in the TSE and QSE, the notched Izod impact strengths were only 100–200 J/m; the exception was the material with one reprocessing cycle through the TSE at 500 rpm (which was 460 J/m). In general, the impact strength decreased with both an increasing number of reprocessing cycles and with higher screw speeds. The Izod impact strengths, however, were similar for the polypropylene reprocessed in the TSE and the QSE. Unlike homopolymer polypropylene, where the Izod impact strength is not greatly affected by heat history [7], this copolymer exhibits substantial decreases with greater reprocessing. This decrease is primarily due to changes occurring in the size of the rubbery phase domains [13,17]. For a single-screw extruder, the impact strength decreased gradually with an increasing number of processing cycles [13]. With a twin-screw extruder, the decrease in impact strength only occurred with higher screw speeds [17]. The differences between prior work and this study could have been due to slight differences in the size of the extruders and/or to differences in the PP copolymers.

### 3.5. Morphology

**Figure 7:**
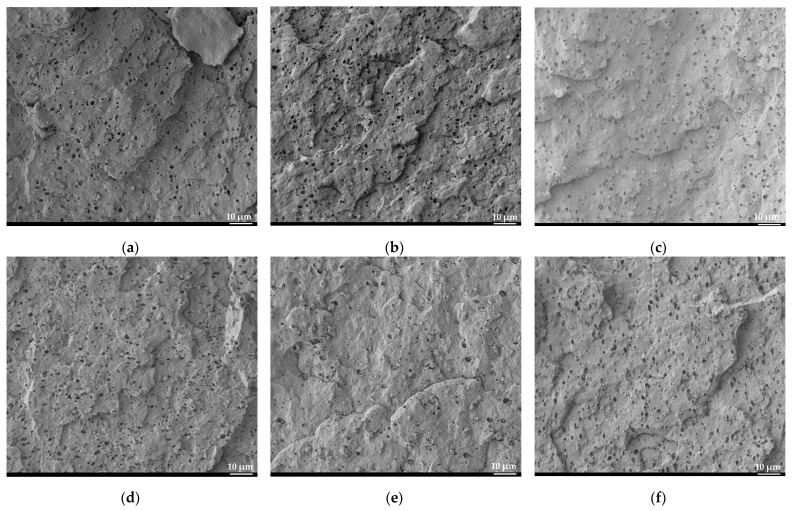

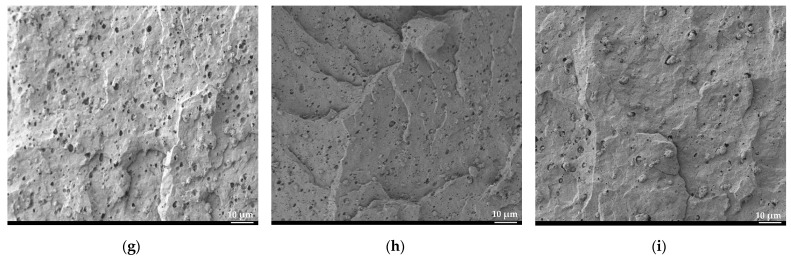
SEM micrographs for (**a**) unprocessed PP; PP reprocessed using a TSE; (**b**) first cycle at 500 rpm; (**c**) third cycle at 500 rpm; (**d**) first cycle at 1500 rpm and (**e**) third cycle at 1500 rpm; and PP reprocessed using a QSE; (**f**) first cycle at 500 rpm; (**g**) third cycle at 500 rpm; (**h**) first cycle at 1500 rpm; and (**i**) third cycle at 1500 rpm.

The SEM micrographs of the cross-section morphology of the unprocessed polypropylene and the polypropylene reprocessed in the TSE and QSE at screw speeds of 500 rpm and 1500 rpm (cycles 1 and 3) are presented in Figure 7. The SEM micrographs revealed a two-phase morphology with spherical shape particles of the ethylene propylene rubber (EPR) minor phase dispersed into the homopolymer polypropylene matrix. The SEM micrograph for the unprocessed polypropylene showed a uniform rubber particle size and particle distribution (Figure 7a). In general, the size and distribution of the dispersed rubbery phase size and distribution was greatly affected by reprocessing. For polypropylene reprocessed in the TSE at 500 rpm (Figure 7b,c), the samples had a smaller particle size and wider particle size distribution, but they were relatively uniformly distributed in the samples. When the screw speed was increased to 1500 rpm in the TSE (Figure 7d,e), the particle size dropped dramatically due to the shearing and breaking of rubber particles into smaller pieces, and the particle distribution was not very uniform. For the samples reprocessed by the QSE at 500 rpm (Figure 7f,i), there was a slight reduction in particle size compared to the unprocessed polypropylene, and the particles were relatively uniformly distributed in the sample. With the higher screw speed of 1500 rpm in the QSE (Figure 7h,i), the same phenomena that occurred with the TSE with higher screw speeds was observed; i.e., the particle size decreased further as the particles broke into smaller pieces due to the shearing effect.

**Figure 8:**
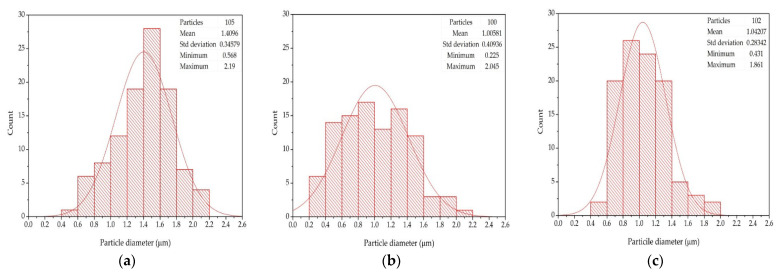
Particle size distribution histograms for (**a**) unprocessed PP, (**b**) first cycle of PP reprocessed using a TSE at 500 rpm, and (**c**) first cycle of PP reprocessed using a QSE at 500 rpm.

To quantify the drop in particle size and the changes in particle size distribution, the micrographs for the unprocessed PP and the first cycle of reprocessed samples by TSE and QSE at 500 rpm were analyzed particle size and particle size distribution using an image processing and analysis software (ImageJ). The particle size distribution was determined by using OriginPro 8.1 software. As shown in Figure 8a, the average particle size from the unprocessed polypropylene was 1.4 µm, and the range of particle sizes was 0.4–2.2 µm. The polypropylene reprocessed at 500 pm in the TSE had a smaller average particle size (1.0 µm) and a slightly wider but less Gaussian particle size distribution (Figure 8b). When the polypropylene reprocessed at 500 pm in the QSE, however, the average particle size was 1.0 µm, and particle size distribution was narrower (Figure 8c).

Similar findings have previously been reported for impact copolymer polypropylene and related copolymers. For a twin-screw extruder operated at 300 rpm, Bouaziz et al. [17] reported a reduction in EPR particle size with an increasing number of reprocessing cycles. Wang et al. [29] also reported a reduction in the domain size for polypropylene/ethylene–octene copolymer blends reprocessed using single-screw extruder. In contrast, Tochacek et al. [13] reported no changes in the average rubber phase size for impact copolymer polypropylene reprocessed using a single-screw extruder, whereas by Bahlouli et al. [30] found no reduction in the particle size for impact copolymer polypropylene reprocessed using a twin-screw extruder.

Interestingly, with this work, the changes particle size and particle distribution seem to correlate with the Izod impact strength results. The highest impact strength was associated with the unprocessed polypropylene, which had the larger EPR particles and a Gaussian particle size distribution. The somewhat lower impact strength correlated with the smaller EPR particles with flat particle size distribution (produced with the TSE at 500 rpm). Finally, the impact strengths of 100–200 J/m were typical for the samples with the smaller particles having a narrow particle size distribution (such as was produced by processing the polypropylene with a QSE at 500 rpm).

## 4. Conclusions

In this work, a heterophasic copolymer polypropylene was reprocessed three times at different screw speeds (500, 1000, and 1500 rpm) in a quad-screw extruder (QSE) and a comparable co-rotating twin-screw extruder (TSE). Both extruders showed higher shear with increased screw speeds. For both the TSE and QSE, increasing the number of reprocessing cycles and the screw speed resulted in higher melt flow indices, decreases in zero-shear viscosity, and shifting of the crossover points for the storage and loss moduli, which indicate a reduction in the molecular weight and a narrowing of the molecular weight distribution. From these measurements, the QSE exhibited greater reductions in molecular weight compared to the TSE due to the higher shear stresses associated the three intermeshing points along the screws. Reprocessing using the TSE and QSE caused a significant reductions in the Izod impact strength of the reprocessed polypropylene. SEM imaging and subsequent analyses showed reductions in the particle size and particle size distribution of the dispersed rubbery phase size in the polypropylene during reprocessing. These changes in the morphology of the dispersed rubbery phase correlated with reductions in the Izod impact strength of the polypropylene samples. Compared to the TSE, the QSE exhibited a higher melt temperatures due to the higher shear stresses and a lower head pressure because of the higher free volume. As a result of changes in polypropylene viscosity, the melt temperature and the head pressure decreased as the number of reprocessing cycles was increased.

## Figures and Tables

**Table 1:** Crossover point (G*) and angular frequency (ω*) at G’ = G” for the PP samples reprocessed using the TSE and QSE.

	Screw Speed	No. of Cycles	G*	ω*
	rpm		kPa	Hz
Unprocessed PP	-	-	27.8	4.0
TSE	500	1	29.4	5.6
		2	29.7	6.7
		3	30.7	9.2
	1000	1	30.6	6.6
		2	34.3	11.3
		3	34.3	15.2
	1500	1	32.7	9.0
		2	-	>15.92
		3	-	>15.92
QSE	500	1	28.5	6.2
		2	30.6	7.9
		3	32.4	8.8
	1000	1	30.4	7.7
		2	30.8	11.8
		3	-	>15.92
	1500	1	33.0	11.6
		2	-	>15.92
		3	-	>15.92

## Data Availability

The data presented in this study are available on request from the corresponding author.
